# Clinical Associations and Coexistence of Polyomavirus DNAemia with EBV and CMV in Pediatric Hematology/Oncology Patients, Including HCT Recipients—A Pilot Study

**DOI:** 10.3390/pathogens14111122

**Published:** 2025-11-04

**Authors:** Tomasz Bogiel, Mateusz Rzepka, Dagmara Depka-Radzikowska, Patrycja Zalas-Więcek, Krzysztof Czyżewski, Monika Richert-Przygońska, Jan Styczyński, Robert Dębski, Elżbieta Grześk, Grzegorz Grześk, Piotr Kanarek, Agnieszka Krawczyk

**Affiliations:** 1 Department of Propaedeutics of Medicine and Infection Prevention, Ludwik Rydygier Collegium Medicum in Bydgoszcz, Nicolaus Copernicus University in Toruń, 85-094 Bydgoszcz, Poland; 2 Clinical Microbiology Laboratory, Dr. Antoni Jurasz University Hospital No. 1, 85-094 Bydgoszcz, Poland; dagmaradepka@cm.umk.pl (D.D.-R.);; 3 Department of Microbiology, Ludwik Rydygier Collegium Medicum in Bydgoszcz, Nicolaus Copernicus University in Toruń, 85-094 Bydgoszcz, Poland; 4 Department of Paediatrics, Haematology, Oncology, Immunology and Transplantology, Ludwik Rydygier Collegium Medicum in Bydgoszcz, Nicolaus Copernicus University in Toruń, 85-094 Bydgoszcz, Polandjstyczynski@cm.umk.pl (J.S.); ellag@cm.umk.pl (E.G.); 5 Clinic of Pediatric, Hematology and Oncology, Dr. Antoni Jurasz University Hospital No. 1, 85-094 Bydgoszcz, Poland; 6 Department of Cardiology and Clinical Pharmacology Ludwik Rydygier Collegium Medicum in Bydgoszcz, Nicolaus Copernicus University in Toruń, 85-168 Bydgoszcz, Poland; g.grzesk@cm.umk.pl; 7 Department of Microbiology and Plant Ecology, Faculty of Agriculture and Biotechnology, Bydgoszcz University of Science and Technology, 85-029 Bydgoszcz, Poland; 8 Department of Molecular Medical Microbiology, Chair of Microbiology, Jagiellonian University Medical College, 31-007 Cracow, Poland; agnieszka1.krawczyk@uj.edu.pl

**Keywords:** BKPyV, HCT, hematology, hematopoietic cell transplantation, herpesviruses, immunocompromised patients, JCPyV, leukemias, oncology, opportunistic infections, viremia

## Abstract

Polyomaviruses (BKPyV and JCPyV) and herpesviruses (EBV, CMV) usually infect people during childhood, and may be associated, in some clinical states, with immunocompromised individuals. The aim of this study was to investigate the occurrence and coexistence of polyomavirus (BKPyV and JCPyV) and herpesvirus (CMV and EBV) DNAemia in pediatric hematology/oncology patients, including HCT recipients, and to assess the clinical relevance of polyomaviruses DNAemia. Whole blood samples of 99 children (including 71 patients undergoing HCT) were analyzed for the DNA of the herpes- and polyomaviruses. Co-existence of herpesvirus DNAemia was checked for the patients and clinically analyzed in detail, especially for those positive for BKPyV DNA. BKPyV DNAemia was detected in 15 (15.2%) patients, with viral loads ranging from 1.2 × 10^3^–1.7 × 10^7^ DNA IU/mL. No JCPyV DNA was detected in any of the samples. Coinfections with EBV or CMV DNAemia were observed in a subset of BKPyV-positive patients. BKPyV DNAemia was more frequent among children with leukemia and in those undergoing HCT. Our findings highlight the clinical associations between BKPyV and herpesvirus DNAemia in immunocompromised pediatric patients. Routine BKPyV DNA monitoring, alongside standard herpesvirus screening, may provide clinically valuable insights in high-risk pediatric cohorts, particularly those with hematologic malignancies and post-HCT status.

## 1. Introduction

Polyomaviruses are ubiquitous, non-enveloped, double-stranded DNA viruses belonging to the *Polyomaviridae* family. BK polyomavirus (BKPyV) and JC polyomavirus (JCPyV) are the most commonly known representatives, initially described in the 1970s, although additional human polyomaviruses, such as Merkel cell polyomavirus, WU polyomavirus, and KI polyomavirus, have since been identified [1,2]. For over three decades, these polyomaviruses were the only thoroughly studied human polyomaviruses. However, the rapid advancement of modern molecular techniques (e.g., next-generation sequencing) has led to a significant increase in the detection of new polyomaviruses [3].

BKPyV and JCPyV infections typically occur in childhood, are mostly asymptomatic, and the viruses usually reside latently in the kidneys, brain, peripheral blood, or urothelium. Therefore, a majority of adults are seropositive, as well as latently infected. However, when reactivated or acquired in the immunocompromised host, BKPyV and JCPyV have been implicated in some human clinical disease states [1]. The reactivation of infection with both viruses may be connected to the applied immunosuppressive therapy or to the general immunodeficiency of the individuals [4,5,6,7,8,9].

BKPyV is of particular clinical relevance in pediatric patients undergoing hematopoietic cell transplantation (HCT), as infection can lead to substantial morbidity and mortality. The virus is most commonly associated with renal complications, including ureteral stenosis, hemorrhagic cystitis (HC), and nephropathy. BKPyV seroprevalence reaches up to 90% in adults, with antibodies most frequently detected among adolescents and young adults [5,9,10]. Despite its high clinical incidence, clinically relevant disease is largely confined to immunocompromised populations, including transplant recipients and children receiving chemotherapy [11]. A major cause of morbidity and mortality following allogeneic HCT (allo-HCT) is virus-associated HC. Less commonly, it is associated with pneumonitis, retinitis, liver disease, and meningoencephalitis [5,8,12]. BKPyV infection is infrequently related to disease amongst healthy individuals; in immunosuppressed counterparts, reactivation could be one of the causes of renal (BKPyV-associated nephropathy, BKPyV-AN) or bladder (HC and ureteral stenosis) damage. Of note, it may also afflict acute lymphoblastic leukemia-suffering patients who developed BKPyV-associated HC without HCT on standard maintenance chemotherapy [12] or who may be accompanied with tubulointerstitial nephritis [13]. It was also shown that BKPyV may also lead to nephropathy in pediatric HCT (auto-HCT) recipients [14].

JCPyV, highly homologous to BKPyV, is primarily associated with progressive multifocal leukoencephalopathy (PML), a demyelinating and often fatal disease of the central nervous system [15,16]. PML is found amongst individuals treated with immunomodulatory therapies as a consequence of confirmed underlying immunodeficiency. JCPyV is also possibly implicated in the development of the other various human neoplasms [4,7,8]. Numerous studies have investigated the oncogenic potential and migration of JCPyV, employing transgenic animal models for these investigations [17].

Herpesviruses, such as cytomegalovirus (CMV) and Epstein–Barr virus (EBV), are another major cause of morbidity in immunocompromised children after HCT. Both viruses are routinely monitored in clinical practice, as their reactivation is associated with life-threatening complications and high treatment costs [18,19]. By contrast, the routine detection of polyomaviruses is not currently standard in many transplant centers, despite evidence that BKPyV reactivation significantly contributes to adverse outcomes. The co-existence of herpesviruses and polyomaviruses in pediatric patients has not been thoroughly investigated. Reports addressing the simultaneous monitoring of CMV, EBV, BKPyV, and JCPyV are rare and largely limited to small cohorts or adult populations [20,21,22]. This gap is especially important in pediatric patients, who are at higher risk of multiple viral reactivations due to immunosuppression caused by both their underlying disease and its treatment.

The aim of this study was to investigate the occurrence and coexistence of polyomavirus (BKPyV and JCPyV) and herpesvirus (CMV and EBV) DNAemia in pediatric hematology/oncology patients, including HCT recipients. We further aimed to assess the clinical relevance of polyomaviruses DNAemia and its potential association with co-infections, disease severity, and post-transplant complications, to evaluate whether routine monitoring of BKPyV/JCPyV DNA could be clinically justified in this population. We hypothesized that herpesvirus infection or reactivation (CMV or EBV) may coincide with or predispose to BKPyV or JCPyV DNAemia, reflecting shared mechanisms of viral reactivation under immune suppression. Furthermore, based on previous reports, we hypothesized that BKPyV, rather than JCPyV, may play a more prominent role in clinical manifestations among immunocompromised pediatric patients, particularly those undergoing HCT or intensive chemotherapy.

There are no specific antiviral therapies for BKPyV and JCPyV infection so far, with immunosuppression reduction being the most commonly applied therapy; therefore, there is a need for further research [7,23]. The need to focus on the mentioned polyomaviruses has recently been discussed in the context of reasonable and reliable diagnostic procedures, as well as possible treatment options for the infections they cause.

## 2. Materials and Methods

This study included 99 patients (60 male and 39 female; age range: 28 days–20 years), all treated in the Clinic of Pediatric, Hematology, and Oncology with in the Hematopoietic Cell Transplant Unit of University Hospital No. 1 in Bydgoszcz, Poland. The blood samples from all of the patients hospitalized in the mentioned unit in the study period were collected at a single time point in case of one the following indications: (i) existing at the time of initial diagnosis, (ii) simultaneous to investigations of herpesviruses DNA made weekly according to the routine clinical diagnostic schemes applied for pediatric post-transplantation patients, or (iii) clinical symptoms of infections observed during hospitalization.

The samples were collected between 2019 and 2022 and analyzed for BKPyV and JCPyV DNA presence (*n* = 99). The characteristics of the patients included in this study are presented in Table 1. Of note, BKPyV/JCPyV DNA status was also checked in two urine samples, derived simultaneously with blood, from two patients at the time of BKPyV DNAemia (data mentioned below).

DNA was extracted separately for each whole blood sample (of note, urine was extracted for two particular samples) with the use of GeneProof Pathogen Free DNA Isolation Kit (GeneProof, Brno, Czech Republic), IVD certified and dedicated for clinical samples.

**Figure 1:**
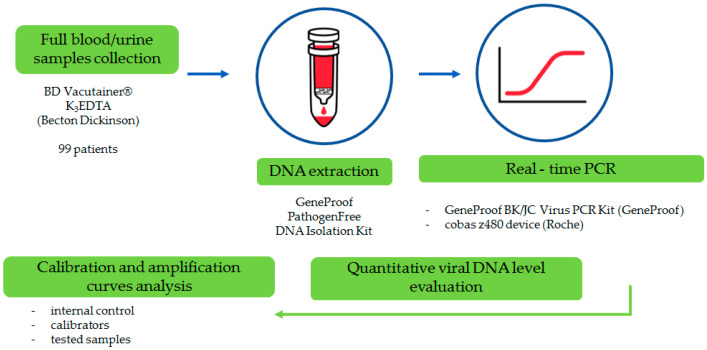
Study workflow.

BKPyV and JCPyV DNA was detected using the certified GeneProof BK/JC Virus (BK/JC) PCR Kit (GeneProof, Brno, Czech Republic), based on a quantitative real-time PCR using a cobas z480 device (Roche, Basel, Switzerland). The results of viral DNA presence were calculated and expressed as international unit (IU) per milliliter (IU/mL) of the tested sample. The study workflow is presented in Figure 1.

Both procedures were performed according to the manufacturer’s recommendations. The manufacturer declares an analytical sensitivity (limit of detection at 95% probability) of 0.684 copies/µL, a linear range from 10^2^ to 10^10^ copies/mL with a precision of ±0.5 log, and 100% specificity for BK and JC viruses [24].

To check the reliability of the applied methodology and confirm the most probable BKPyV DNA viremia for 2 of these 15 patients (patient No. 1 and 7, Table 2) the BKPyV status of urine sample was also investigated prospectively at the time of observation, with outstanding levels of viral DNA in urine reaching 8.4 × 10^10^ and 1.1 × 10^10^ IU/mL, respectively.

Cytomegalovirus (CMV) and Epstein–Barr (EBV) viremia incidents were additionally analyzed retrospectively for all of the patients included in this study. The testing for both viral DNA was performed with the application of the appropriate GeneProof CMV PCR Kit and GeneProof EBV PCR Kit (GeneProof, Brno, Czech Republic), respectively. All procedures were performed according to the manufacturer’s instructions. The manufacturer declares, for the CMV PCR Kit, an analytical sensitivity (limit of detection at 95% probability) of 122.594 IU/mL, a linear range from 10^2.5^ to 10^10^ IU/mL with precision of ±0.5 log, and an analytical specificity of 100% for CMV (diagnostic specificity 90.67%, CI_95%_: 81.15–95.85%) [25]. For the EBV PCR Kit, the manufacturer declares an analytical sensitivity of 196.1 IU/mL for plasma, 129.2 IU/mL for whole blood, and 110.8 IU/mL for CSF, a linear range from 10^2.5^ to 10^10^ IU/mL with precision of ±0.5 log, and an analytical specificity of 100% for EBV (diagnostic specificity 94.19%; CI_95%_: 86.35–97.84%) [26].

Data interpretation and statistical analysis was performed using the Statistica™ 13.3 (TIBCO Software Inc., Palo Alto, CA, USA) program. Statistical calculations were performed with the application of chi-square test for BKPyV-positive results with respect to patients’ gender, diagnosis, application of HCT, and graft-versus-host disease (GvHD), exclusively for the patients undergoing HCT, to investigate statistically significant differences between the groups. If the assumptions of the chi-square test were not met, Fisher’s exact two-tailed test was used. Differences at the level of *p* < 0.05 were considered statistically significant.

## 3. Results

In total, 15 (15.2%) out of 99 whole blood samples were positive for BKPyV DNA, with the range of viral load of 1.2 × 10^3^–1.7 × 10^7^ IU/mL. It should be noted that only a single blood sample per participant was analyzed, which limits the assessment of temporal dynamics of viral reactivation and prevents capturing the onset, peak, and resolution of BKPyV and JCPyV DNAemia. However, CMV and EBV viremia were additionally analyzed retrospectively from clinical records, providing a broader view of herpesvirus activity despite the single-time-point sampling for polyomaviruses. The detailed characteristics of patients with BKPyV DNAemia are presented in Table 2.

**Figure 2:**
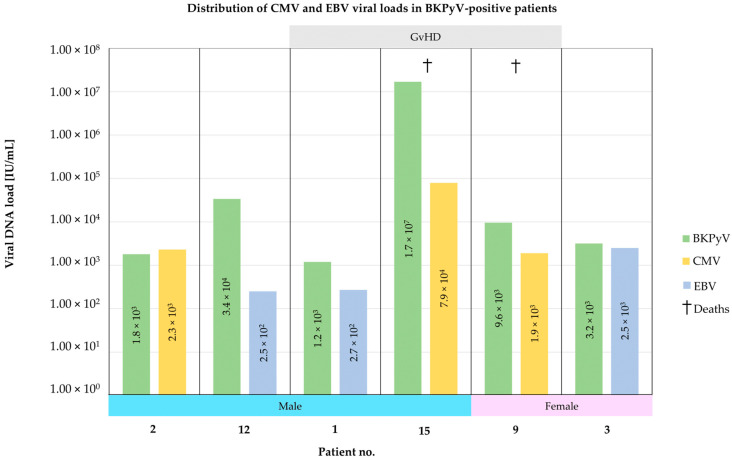
Distribution of CMV or EBV DNA levels [IU/mL] in the whole blood samples with BKPyV (green) DNA presence with respect to patient’s gender, complications, and outcome (mortality). CMV—cytomegalovirus (yellow); EBV—Epstein–Barr virus (blue); GvHD—graft-versus-host disease; IU—international unit.

Among the 15 BKPyV-positive patients, 14 (93.3%) had previously undergone HCT and received methotrexate and cyclosporine GvHD-preventing treatment. As has been shown (Figure 2), three (20.0%) of them presented simultaneous CMV DNA presence (CMV DNA viral load of 1.9 × 10^3^, 2.3 × 10^3^ and 7.9 × 10^4^ IU/mL, respectively). For the other three (20.0%) patients, EBV DNA was detected at the same time (at the levels of 2.5 × 10^2^, 2.7 × 10^2^ and 2.5 × 10^3^ IU/mL, respectively). Thus, approximately 40% of BKPyV-positive patients had concurrent herpesvirus DNAemia.

Amongst the five BKPyV-positive patients (No. 1, 5, 7, 9, and 15, Table 2) who died in the study period, two (patients No. 9 and 15) also presented CMV DNAemia. The characteristics and distributions of BKPyV DNA-positive patients, in terms of results positive for herpesviruses co-infections, are presented in Figure 2.

Patients with positive BKPyV results (fifteen altogether, nine male and six female; age range: 4.1–12.9 years) presented diverse symptoms (eleven patients) or were asymptomatic (four patients) at the sample collection date (for details, see Table 2).

It is noteworthy that, out of the four asymptomatic patients, one (patient No. 8) exhibited a relatively high level of BKPyV DNA (1.4 × 10^4^ IU/mL), but had not undergone HCT at the time of the research.

In the context of antiviral treatment, patient No. 11 (from Table 2) was receiving cidofovir treatment during the study period, one BKV-negative but CMV-positive (1.04 × 10^4^ IU/mL) was receiving ganciclovir, and one more patient, BKV-negative and CMV-positive (8.7 × 10^3^ IU/mL), was receiving both ganciclovir and cidofovir.

None of the blood samples were positive for JCPyV DNA.

In the overall analysis of herpesvirus infections amongst the whole BKPyV study group (*n* = 99), CMV DNA was noted for twelve (12.1%) patients in the studied group (CMV DNAemia in the range 1.9 × 10^3^–7.9 × 10^4^ IU/mL), while EBV DNA cases were observed among nine (9.1%) patients, with EBV DNAemia in the range of 2.5 × 10^2^–2.5 × 10^3^ IU/mL.

It should be noted that these values reflect the prevalence in the entire study cohort, complementing the subgroup data described above.

No statistically significant differences were observed in the BKPyV-positive DNAemia between the groups of patients in terms of applied HCT (χ^2^ = 0.04114, *p* = 0.0602), gender (χ^2^ < 0.001, *p* = 0.9584), and GvHD diagnosis (calculated only for HCT-undergoing patients, χ^2^ = 1.35, *p* = 0.2461). However, BKPyV-positive results were noted significantly more often in the group of patients suffering from leukemia (all leukemia types altogether, χ^2^ = 0.10657, *p* = 0.0008) than from any other conditions.

## 4. Discussion

Viral infections remain a major source of morbidity and mortality in pediatric patients undergoing hematopoietic cell transplantation [27,28]. In some immunodeficiency conditions, BKPyV can reactivate to cause, e.g., HC and nephritis [29], or even systemic infection [30]. This may explain the extremely high viral DNA loads observed in two of our patients with confirmed BKPyV DNAemia. Afterwards, in the infection pathogenesis, BKPyV reaches bloodstream of the immunocompromised patients. Thus, a high rate of BKPyV viremia infections was observed in the pediatric cohort included in the study. Our findings expand on the current literature by demonstrating that BKPyV viremia occurs in approximately 15% of immunocompromised pediatric patients (most of whom were post-HCT) and that it frequently coexists with CMV or EBV DNAemia. This co-detection suggests that shared mechanisms of immune dysregulation may cause multiple viral reactivations in this vulnerable group, particularly in the context of leukemia or post-transplant immunosuppression [19]. In immunocompromised hosts, impaired T-cell-mediated surveillance, including both CD8+ cytotoxic T lymphocytes and CD4+ helper T cells, may reduce control over latent viral infections [31,32,33]. Reduced activity of virus-specific T cells allows for the simultaneous reactivation of latent viruses such as BKPyV, CMV, and EBV. Furthermore, the use of immunosuppressive agents, such as cyclosporine or methotrexate, can exacerbate this immune impairment, leading to simultaneous viral DNAemia. Such concurrent viral reactivation may contribute to increased morbidity, influence transplant outcomes, and exacerbate complications like graft-versus-host disease or nephropathy, underscoring the importance of integrated viral monitoring in high-risk pediatric populations [19,31,34].

JCPyV was not identified in any of the investigated samples, which indicates that BKPyV DNAemia is more frequently observed than JCPyV DNAemia in this population, highlighting its potential clinical relevance. This observation provides one of the few pediatric datasets directly comparing both polyomaviruses in the same cohort, thereby strengthening the notion that JCPyV plays a limited role in this clinical context. The frequency of BKPyV detection in our cohort aligns with previous studies reporting higher prevalence among pediatric HCT recipients [26,27,28]. However, unlike most earlier studies, which focused solely on post-transplant populations, our analysis also included leukemia patients outside the HCT setting, offering a more comprehensive view of viral epidemiology in immunocompromised children. Analysis performed by Wei et al. revealed that 40.9% of pediatric patients developed BKPyV infection after HCT. In their study, viral load was commonly detected in urine and, in approximately one third of cases, also in blood, with median values of 9.5 × 10^7^ copies/mL and 2.97 × 10^3^ copies/mL, respectively [35]. Notably, the first detection of BKPyV in urine occurred significantly earlier than in blood (median: 13.5 vs. 30.5 days), suggesting that urine monitoring may serve as an early marker of systemic infection. The authors further demonstrated that more than half of BKPyV-infected patients developed hemorrhagic cystitis (59.0%), while nearly one quarter exhibited renal impairment (24.6%). Moreover, BKPyV infection was associated with an increased risk of transplant-associated thrombotic microangiopathy (TA-TMA), veno-occlusive disease (VOD), and diffuse alveolar hemorrhage. Finally, multivariate analysis identified age above five years and the use of mycophenolate mofetil (MMF) as independent risk factors for BKPyV infection after HCT [35]. In contrast to this cohort, our study demonstrates that, even without MMF-based immunosuppression, BKPyV DNAemia may occur, suggesting that other leukemia- or therapy-related immune alterations may similarly predispose to reactivation.

Anther independent study conducted by Salamonowicz-Bodzioch et al. showed that 27% pediatric patients undergoing HCT were positive for BKPyV presence. Moreover, the researchers identified several significant risk factors for BKPyV-HC incidence, including age over five years, matched unrelated donor (MUD) transplantation, busulfan–cyclophosphamid–melphalan conditioning, acute myeloblastic leukemia diagnosis, and the presence of aGVHD [36]. These findings further emphasize that both host-related and treatment-related factors strongly influence the risk of BKPyV complications in pediatric HCT recipients. Our results extend this understanding by demonstrating the simultaneous detection of herpesvirus DNAemia in several BKPyV DNA-positive patients, supporting the hypothesis of shared immune reactivation pathways among latent viruses. In turn, Kaya et al. showed that BKPyV infection was detected in more than half (52.9%) of pediatric patients undergoing HSCT, with positive results observed predominantly among allogeneic recipients (56.5%) compared to those receiving autologous transplantation (20.0%). Importantly, a urinary viral load exceeding 10^7^ copies/mL was identified in 26.1% of allogeneic HSCT patients and was regarded as a level requiring preemptive therapeutic intervention. Furthermore, BKPyV viruria exceeding 10^9^ copies/mL was consistently detected within two weeks prior to HC onset, supporting its role as a prognostic indicator for predictive diagnosis. BKPyV viremia above 10^4^ copies/mL was also reported in a single patient before HC manifestation [37]. Based on these findings, the authors emphasized the clinical utility of routine screening for BKPyV infection, particularly monitoring viruria, as a valuable tool in the early identification and prevention of HC in high-risk pediatric HSCT recipients. Our data reinforce this recommendation and extend it to hematology/oncology patients outside the transplant context, in whom routine testing for BKPyV is rarely performed, but may provide important clinical information.

Giraud et al., in their study of large HCT cohorts, have also shown that BKPyV viruria incidences, often accompanied by HC, are more frequent in a group of allogenic HCT patients achieving full conditioning with HLA-mismatched unrelated grafts. Moreover, BKPyV viruria was more prevalent during HC than in non-HC events, being an independent HC risk factor [38]. These findings support the clinical value of BKPyV viruria testing as a non-invasive prognostic marker.

Furthermore, recent evidence highlights that BKPyV-related complications are not restricted to transplant settings. A study from Hospital in Ankara described 12 pediatric acute leukemia patients, all undergoing intensive chemotherapy without HSCT, in whom BKPyV infection was confirmed by PCR. Most cases (10/12) occurred in children with acute lymphoblastic leukemia, predominantly T-cell ALL, and clinical manifestations were dominated by HC (11/12 cases), and one patient had epididymitis. Male gender and age above 10 years were common features, while extremely high viral loads (up to 1.3 × 10^12^ copies/mL) were observed. Importantly, nearly all patients improved following supportive or symptomatic therapy, except one with refractory disease. These findings suggest that, similar to HSCT recipients, older male patients and those with profound T-cell dysfunction may be at particular risk of BKPyV reactivation [39]. Our findings are consistent with these observations, and strengthen the concept that BKPyV may act as a clinically relevant pathogen in pediatric hematology, independent of transplantation—an area that remains underexplored.

It is worth noting that the prevalence of polyomavirus infections varies depending on the tested population and the diagnostic method used [40]. Therefore, utilizing data on the genetic variability of both viruses proves to be highly beneficial, primarily for those predominant in Europe and especially at the current research facility [41,42,43]. In this context, our study provides valuable epidemiologic data on the co-occurrence of BKPyV, CMV, and EBV in pediatric patients after hematopoietic cell transplantation in Poland, addressing a gap in similar analyses in this population.

In our study, among the 15 patients with BKPyV DNAemia, the majority (93.3%) had previously undergone HCT and received methotrexate and cyclosporine for GvHD prophylaxis. Notably, co-detection of CMV DNA was observed in 3 patients (20.0%), with viral loads ranging from 1.9 × 10^3^ to 7.9 × 10^4^ IU/mL, while EBV DNA was detected in another 3 patients (20.0%) at levels from 2.5 × 10^2^ to 2.5 × 10^3^ IU/mL. Importantly, two of the five BKPyV-positive patients who died during the study period also presented concomitant CMV DNAemia. In the BKPyV-positive patients (*n* = 15), three had CMV DNAemia and three had EBV DNAemia, corresponding to approximately 40% of BKPyV-positive cases. Such viral co-existence may exacerbate immune dysregulation, contribute to a range of treatment-related complications, and further impair antiviral responses, thereby worsening clinical outcomes [44,45]. In the context of the entire cohort (*n* = 99), CMV was documented in twelve patients (12.1%) and EBV in nine patients (9.1%), highlighting the tendency of these viruses DNA presence in the setting of weakened host immunity.

The observation that two of the five deceased patients had concomitant BKPyV and CMV DNAemia may suggest a possible synergistic negative impact of these viruses. CMV infection itself is associated with increased mortality and transplant-related complications, and the concomitant presence of BKPyV could potentially further influence the course of the disease, particularly within the urinary tract and kidneys [20,46]. These observations raise the hypothesis that polyomavirus DNAemia may be an additional factor contributing to morbidity in children with prior herpes virus reactivation, although a causal relationship cannot be established based on our data. This novel association highlights the potential value of including BKPyV into multiplex viral monitoring protocols, particularly in patients who already have herpesvirus viremia.

Furthermore, the detection of high levels of BKPyV DNA in an asymptomatic patient highlights that viremia does not always translate into overt clinical symptoms. However, such cases may still be associated with a risk of subclinical organ involvement or may represent an early stage preceding clinical manifestations. Therefore, close monitoring of these patients may be warranted to prevent progression to symptomatic disease. This subclinical detection adds a new dimension to the understanding of BKPyV DNAemia dynamics, suggesting that early viremia may precede clinical disease and warrant preemptive observation. Finally, the relatively noticeable frequency of BKPyV detection among patients with leukemia as observed in the studies discussed above and confirmed in our results, indicates that disease-related immune perturbations, combined with intensive chemotherapy, predispose this population to viral infections or their reactivation [20,32,35,36,37,47]. This highlights the clinical relevance of BKPyV monitoring not only in post-HCT patients, but also in selected groups of pediatric oncology patients receiving high-intensity treatment.

To the best of our knowledge, the only studies addressing simultaneous CMV, EBV, BKPyV, and JCPyV DNA investigation in the clinical material samples derived from immunocompromised patients was conducted by Rota et al. [48] and Rahiala et al. [49]. The results of the first research are slightly similar to those of the present study. It is underlined that especially medical centers performing bone marrow or kidney transplantation should introduce procedures linked to BKPyV/JCPyV infections or their reactivations to routine diagnostic schemes (initial diagnostic and viral DNA status checking), since these infections of this origin are crucial in immunosuppressive patients [50,51].

The research performed by Rota et al. [48] among immunocompromised patients revealed that blood sample of only one (1.6%) patient was positive for BKPyV DNA. Meanwhile, JCPyV DNA was not detected in any of the samples included in the study. The last observation is consistent with the results of our study. Meanwhile, Delbue et al. detected BKPyV and JCPyVDNA in a cerebrospinal fluid samples among 6.4% and 1.3% of the patients included in their study, respectively [52]. However, the latter research was not restricted to pediatric patients. Thus, the only patient in the cited study with blood-positive BKPyV DNA status was a kidney transplant recipient [48]. In contrary, using standard and real-time PCR, the study conducted by Whiley et al. revealed positive results of polyomaviruses DNA amongst 40.2% [53] and 35.4% [54] of the urine samples of the patients included in their study, respectively.

To summarize, relatively little is still known about the epidemiology and clinical characteristics of BKPyV and JCPyV in immunocompromised pediatric patients, particularly those with hematologic disorders. Our findings confirm that JCPyV DNAemia is rather uncommon in this group, while BKPyV detection may provide clinically useful insights, especially in patients presenting with unclear infectious symptoms. These results highlight a potential value of monitoring BKPyV and JCPyV DNA in selected high-risk pediatric cohorts [47,49,55], in a manner comparable to herpesvirus surveillance such as CMV, without implying a direct causal relationship or universal screening recommendation.

The main limitations of our study include the relatively small and heterogeneous sample size and the lack of simultaneous testing in both urine and blood, which may have limited our ability to fully assess viral dynamics. Moreover, only a single blood sample per participant was collected, precluding the assessment of the temporal dynamics of BKPyV and JCPyV, including the onset, peak, and resolution of viral reactivation. CMV and EBV viremia were analyzed retrospectively from patient records, providing some insight into herpesvirus DNA presence, despite the single-time-point sampling for polyomaviruses. Consequently, while our data provide valuable information on the prevalence and co-occurrence of these viruses, they cannot fully capture the course of infection over time. Notably, for two patients with confirmed BKPyV DNAemia in blood, simultaneous urine samples were analyzed and revealed very high viral loads, supporting the reliability of the blood-based detection and demonstrating that, even at a single time point, clinically relevant viremia can be identified. Despite these limitations, our study provides one of the first systematic assessments of concurrent BKPyV, JCPyV, CMV, and EBV DNAemia in a pediatric cohort, establishing a foundation for future longitudinal investigations.

Further studies in larger pediatric populations are warranted to better define the interplay between immunosuppression, immune competence, and BKPyV infection or its reactivation. Such data will be crucial for refining surveillance strategies and developing standardized diagnostic and management approaches for polyomavirus infection risk in immunocompromised children. Taken together, our findings add to the limited body of literature regarding issues of opportunistic viruses in pediatric populations with blood disorders, including those undergoing immunosuppressive treatments.

Although this study provides observational findings highlighting the importance of BKPyV in this patient cohort, further research is planned to not only correlate BKPyV incidence with other opportunistic pathogens, but also to support the routine inclusion of BKPyV diagnostics in clinical screening. More robust analyses in larger cohorts would help us to establish standardized approaches, similar to those used for kidney transplantation patients.

## 5. Conclusions

BKPyV DNAemia was detected in approximately 15.2% of pediatric immunocompromised patients, often co-existing with herpesvirus (CMV, EBV) DNAemia. JCPyV DNAemia was not observed in this cohort. These findings highlight a potential association between BKPyV and herpesvirus DNAemia in high-risk pediatric patients, suggesting that BKPyV monitoring could provide useful observational insights, particularly in children with leukemia or after HCT, without implying a universal screening recommendation.

## Figures and Tables

**Table 1:** Primary diagnosis of the patients included in the study (*n* = 99); the most common conditions/immunodeficiencies are specified.

Diagnosis	Patients (*n* = 99)		HCT Patients (*n* = 70)	
	*n*	%	*n*	%
Acute lymphoblastic leukemia (ALL)	46	46.5	38	54.3
Acute myeloid leukemia (AML)	16	16.2	15	21.4
Aplastic anemia (acquired or congenital, inc. Blackfan-Diamond)	9	9.1	7	10.0
Inborn errors of immunity (IEI, includingbone marrow failure)	4	4.0	3	4.3
Immune thrombocytopenia	4	4.0	0	0.0
Hemophagocytic syndrome (HLH)	3	3.0	1	1.4
Myelo dysplastic syndrome (MDS)	2	2.0	2	2.9
Congenital or acquired CMV infection	2	2.0	0	0.0
Non-Hodgkin-lymphoma (NHL)	1	1.0	1	1.4
Neuroblastoma	1	1.0	1	1.4
Nejmegen syndrome (NBS)	1	1.0	1	1.4
Chronic granulomatous disease (CGD)	1	1.0	1	1.4
Cerebellar syndrome	1	1.0	0	0.0
Splenic abscesses	1	1.0	0	0.0
Rhabdomyosarcoma (RML)	1	1.0	0	0.0
Monocytosis	1	1.0	0	0.0
Wilms’s tumor	1	1.0	0	0.0
Brain tumor	1	1.0	0	0.0
Bone marrow donor	1	1.0	0	0.0
B-cell lymphoma	1	1.0	0	0.0
Hydrocephalus, neuroinfection, arachnoid cyst	1	1.0	0	0.0

HCT—hematopoietic cells transplantation.

**Table 2:** Characteristics of patients with BKPyV DNAemia (*n* = 15).

Patient No.	Diagnosis	Viral DNA Level [IU/mL]			Age at the Time of BKPyV-Positive Result (Years)	Symptoms at the Samples Collection Time	Age at the Time of Death	Direct Reason of Death	Time Between Initial Diagnosis and BKPyV DNA Presence Confirmation (Years)	Time Between HCT and BKPyV DNA Presence Confirmation (Months)
		BKPyV	CMV	EBV						
1	AML	1.2 × 10^3^	0	2.7 × 10^2^	9	fever, dysuria, abdominal pain, lymphoproliferative disease	9 years	multi-focal stroke	3.5	2.7
2	ALL	1.8 × 10^3^	2.3 × 10^3^	0	7	bladder infection	N/A	N/A	3.8	1.8
3	ALL	3.2 × 10^3^	0	2.5 × 10^3^	4	lymph nodes enlargement, EBV PTLD	N/A	N/A	0.8	0.7
4	SAA	9.1 × 10^3^	0	0	14	fever, dysurical symptoms	N/A	N/A	0.5	0.3
5	ALL	4.9 × 10^3^	0	0	12	consciousness disturbances, encephalopathy	12 years	toxic encephalopathy	0.7	1.2
6	ALL	5.2 × 10^3^	0	0	6	fever	N/A	N/A	2.2	0.9
7	AML	5.3 × 10^3^	0	0	4	bladder infection, urinemia	5 years	AML relapse	0.6	1.3
8	ALL	9.1 × 10^3^	0	0	9	asymptomatic *	N/A	N/A	0.8	1.4
9	AML	9.6 × 10^3^	1.9 × 10^3^	0	10	gastrointestinal tract bleeding, fever, leukopenia	10 years	toxic encephalopathy, CMV reactivation	0.8	1.9
10	ALL	1.4 × 10^4^	0	0	12	asymptomatic *	N/A	N/A	1.1	N/A
11 **	ALL	2.2 × 10^4^	0	0	5	hemorrhagic cystitis, mycosis of the left lung	N/A	N/A	1.5	1.0
12	ALL	3.4 × 10^4^	0	2.5 × 10^2^	12	asymptomatic *	N/A	N/A	0.7	0.9
13	ALL	3.7 × 10^4^	0	0	10	fever	N/A	N/A	1.5	9.6
14	AML	2.9 × 10^5^	0	0	9	thrombocytopenia, fever, ALAT level increase	N/A	N/A	3.1	3.3
15	ALL	1.7 × 10^7^	7.9 × 10^4^	0	10	asymptomatic *	11 years	multiorgan failure	1.4	6.5

ALAT—alanine aminotransferase; ALL—acute lymphoblastic leukemia; AML—acute myeloid leukemia; CMV—cytomegalovirus; EBV—Epstein–Barr virus; HCT—hematopoietic cell transplantation; IU—international unit applied to express the standardized viral DNA level); N/A—not applicable; PTLD—post-transplant lymphoproliferative disease; SAA—severe aplastic anemia; *—patients without any obvious viral infection symptoms; **—patient was receiving cidofovir treatment during the study period.

## Data Availability

The original contributions presented in this study are included in the article. Further inquiries can be directed to the corresponding authors.

## Abbreviations

ALAT	alanine aminotransferase
ALL	acute lymphoblastic leukemia
AML	acute myeloid leukemia
CMV	cytomegalovirus
EBV	Epstein–Barr virus
GvHD	graft-versus-host disease
HCT	hematopoietic cell transplantation
IU	international unit applied to express the standardized viral DNA level
N/A	not applicable
PTLD	post-transplant lymphoproliferative disease
SAA	severe aplastic anemia.

## References

[1] Prezioso C., Pietropaolo V. (2021). BK Virus and Transplantation. Viruses.

[2] Moens U., Prezioso C., Pietropaolo V. (2020). Genetic Diversity of the Noncoding Control Region of the Novel Human Polyomaviruses. Viruses.

[3] Furmaga J., Kowalczyk M., Zapolski T., Furmaga O., Krakowski L., Rudzki G., Jaroszyński A., Jakubczak A. (2021). BK Polyomavirus—Biology, Genomic Variation and Diagnosis. Viruses.

[4] Assetta B., Atwood W.J. (2017). The Biology of JC Polyomavirus. Biol. Chem..

[5] Blackard J.T., Davies S.M., Laskin B.L. (2020). BK Polyomavirus Diversity-Why Viral Variation Matters. Rev. Med. Virol..

[6] Dalianis T., Hirsch H.H. (2013). Human Polyomaviruses in Disease and Cancer. Virology.

[7] Kwak E.J., Vilchez R.A., Randhawa P., Shapiro R., Butel J.S., Kusne S. (2002). Pathogenesis and Management of Polyomavirus Infection in Transplant Recipients. Clin. Infect. Dis..

[8] Pinto M., Dobson S. (2014). BK and JC Virus: A Review. J. Infect..

[9] Sroller V., Hamšíková E., Ludvíková V., Vochozková P., Kojzarová M., Fraiberk M., Saláková M., Morávková A., Forstová J., Němečková S. (2014). Seroprevalence Rates of BKV, JCV, and MCPyV Polyomaviruses in the General Czech Republic Population. J. Med. Virol..

[10] Benketira A., Tichit R., Tenenbaum J., Margueritte G., Bernard F. (2005). BK virus infection in a child after an hematopoietic stem cell transplantation. Arch. Pediatr..

[11] Borriello M., Ingrosso D., Perna A.F., Lombardi A., Maggi P., Altucci L., Caraglia M. (2022). BK Virus Infection and BK-Virus-Associated Nephropathy in Renal Transplant Recipients. Genes.

[12] Alavi S., Yazdi M.K., Parvin M., Zohrehbandian F., Azma R. (2013). Haemorrhagic Cystitis Due to BK Virus in a Child with ALL on Standard Chemotherapy without Stem Cell Transplant. Ecancermedicalscience.

[13] Hoefele J., Rüssmann D., Klein B., Weber L.T., Führer M. (2008). BK Virus Induced Nephritis in a Boy with Acute Myeloid Leukaemia Undergoing Bone Marrow Transplantation. NDT Plus.

[14] Sanchez-Pinto L.N., Laskin B.L., Jodele S., Hummel T.R., Yin H.J., Goebel J. (2011). BK Virus Nephropathy in a Pediatric Autologous Stem-Cell Transplant Recipient. Pediatr. Blood Cancer.

[15] Ahye N., Bellizzi A., May D., Wollebo H.S. (2020). The Role of the JC Virus in Central Nervous System Tumorigenesis. Int. J. Mol. Sci..

[16] Butic A.B., Spencer S.A., Shaheen S.K., Lukacher A.E. (2023). Polyomavirus Wakes Up and Chooses Neurovirulence. Viruses.

[17] Del Valle L., Khalili K. (2021). Induction of Brain Tumors by the Archetype Strain of Human Neurotropic JCPyV in a Transgenic Mouse Model. Viruses.

[18] Li S.S., Zhang N., Jia M., Su M. (2022). Association Between Cytomegalovirus and Epstein-Barr Virus Co-Reactivation and Hematopoietic Stem Cell Transplantation. Front. Cell. Infect. Microbiol..

[19] Bateman C.M., Kesson A., Powys M., Wong M., Blyth E. (2021). Cytomegalovirus Infections in Children with Primary and Secondary Immune Deficiencies. Viruses.

[20] Eslami Kojidi M., Shatizadeh Malekshahi S., Jabbari M.R. (2024). The simultaneous presence of active BK, Epstein Barr, and human cytomegalovirus infection and their correlation by host factors in patients suspected of kidney transplant rejection. BMC Infect. Dis..

[21] Levi S., Davidovits M., Alfandari H., Dagan A., Borovitz Y., Bilavsky E., Landau D., Haskin O. (2022). EBV, CMV, and BK viral infections in pediatric kidney transplantation: Frequency, risk factors, treatment, and outcomes. Pediatr. Transplant..

[22] Jauhiainen M.K., Mohanraj U., Lehecka M., Niemelä M., Hirvonen T.P., Pratas D., Perdomo M.F., Söderlund-Venermo M., Mäkitie A.A., Sinkkonen S.T. (2023). Herpesviruses, polyomaviruses, parvoviruses, papillomaviruses, and anelloviruses in vestibular schwannoma. J. Neurovirol..

[23] Dalianis T., Eriksson B.-M., Felldin M., Friman V., Hammarin A.-L., Herthelius M., Ljungman P., Mölne J., Wennberg L., Swartling L. (2019). Management of BK-Virus Infection—Swedish Recommendations. Infect. Dis..

[24] GeneProof GeneProof BK/JC Virus (BK/JC) PCR Kit. https://www.geneproof.com/geneproof-bk-jc-virus-bk-jc-pcr-kit/p1121.

[25] GeneProof GeneProof Cytomegalovirus (CMV) PCR Kit (IVDR). https://www.geneproof.com/geneproof-r-cytomegalovirus-cmv-pcr-kit-ivdr/p6912.

[26] GeneProof GeneProof Epstein-Barr Virus (EBV) PCR Kit. https://www.geneproof.com/geneproof-epstein-barr-virus-ebv-pcr-kit/p1083.

[27] Admiraal R., de Koning C.C.H., Lindemans C.A., Bierings M.B., Wensing A.M.J., Versluys A.B., Wolfs T.F.W., Nierkens S., Boelens J.J. (2017). Viral reactivations and associated outcomes in the context of immune reconstitution after pediatric hematopoietic cell transplantation. J. Allergy Clin. Immunol..

[28] Lau C.E., DiTullio D.J., Wilhalme H., Bowles L., Moore T.B., De Oliveira S.N. (2025). Prevalence of Viral Infections and Serious Complications in Pediatric Hematopoietic Stem Cell Transplant Patients: A Ten-Year Single-Institution Retrospective Study. J. Hematol..

[29] Ersoy G.Z., Bozkurt C., Aksoy B.A., Öner Ö.B., Aydoğdu S., Çipe F., Sütçü M., Özkaya O., Fışgın T. (2022). Evaluation of the Risk Factors for BK Virus-Associated Hemorrhagic Cystitis in Pediatric Bone Marrow Transplantation Patients: Does Post-Transplantation Cyclophosphamide Increase the Frequency?. Pediatr. Transplant..

[30] Espinosa-González R., León D.E.A., Rodríguez-Jurado R., Uribe-Uribe N.O. (2020). Systemic BK Virus Infection in a Pediatric Patient With Severe Combined Immunodeficiency. Pediatr. Dev. Pathol..

[31] Degli-Esposti M.A., Hill G.R. (2022). Immune control of cytomegalovirus reactivation in stem cell transplantation. Blood.

[32] Kervevan J., Chakrabarti L.A. (2021). Role of CD4+ T Cells in the Control of Viral Infections: Recent Advances and Open Questions. Int. J. Mol. Sci..

[33] Diaz-Salazar C., Sun J.C. (2020). Coordinated Viral Control by Cytotoxic Lymphocytes Ensures Optimal Adaptive NK Cell Responses. Cell Rep..

[34] Karantanos T., Kim H.T., Tijaro-Ovalle N.M., Li L., Cutler C., Antin J.H., Ballen K., Marty F.M., Tan C.S., Ritz J. (2019). Reactivation of BK virus after double umbilical cord blood transplantation in adults correlates with impaired reconstitution of CD^4+^ and CD^8+^ T effector memory cells and increase of T regulatory cells. Clin. Immunol..

[35] Wei A., Jing Y., Zhu G., Wang B., Yang J., Jia C., Luo Y., Yan Y., Zheng J., Zhou X. (2024). Analysis of BK Virus Infection in Children After Hematopoietic Cell Transplantation: A Retrospective Single-center Study. J. Pediatr. Hematol./Oncol..

[36] Salamonowicz-Bodzioch M., Frączkiewicz J., Czyżewski K., Zając-Spychała O., Gorczyńska E., Panasiuk A., Ussowicz M., Kałwak K., Szmit Z., Wróbel G. (2021). Prospective Analysis of BKV Hemorrhagic Cystitis in Children and Adolescents Undergoing Hematopoietic Cell Transplantation. Ann. Hematol..

[37] Kaya N.N., Bayram İ., Öztürk G., Sezgin G., Küpeli S., Yarkın F. (2020). BK Virus Infections in Pediatric Patients with Hematopoietic Stem Cell Transplantation. Duzce Med. J..

[38] Giraud G., Priftakis P., Bogdanovic G., Remberger M., Dubrulle M., Hau A., Gutmark R., Mattson J., Svahn B.M., Ringden O. (2008). BK-viruria and haemorrhagic cystitis are more frequent in allogeneic haematopoietic stem cell transplant patients receiving full conditioning and unrelated-HLA-mismatched grafts. Bone Marrow Transplant..

[39] Kaçar D., Guzelcik Z., Yozgat A.K., Isik M., Yarali N. (2023). BK-virus infections in pediatric leukemia patients during leukemia treatment. Hematol. Transfus. Cell Ther..

[40] da Costa S.d.S.V.Á., Monteiro J.C., Viegas A.P.d.V., de Sá K.S.G., da Cruz S.R., Lima S.S., Vallinoto I.M.V.C., Costa I.B., Vallinoto A.C.R. (2023). Prevalence of JC and BK Polyomavirus Infection in Patients with Chronic Kidney Disease in the State of Pará, Brazil. Trop. Med. Infect. Dis..

[41] Karimi Dehcheshmeh L., Makvandi M., Timori A. (2020). Prevalence of Human Polyomavirus JC and BK in Normal Population. Asian Pac. J. Cancer Prev..

[42] Umeda K., Kato I., Kawaguchi K., Tasaka K., Kamitori T., Ogata H., Mikami T., Hiramatsu H., Saito R., Ogawa O. (2018). High Incidence of BK Virus-Associated Hemorrhagic Cystitis in Children after Second or Third Allogeneic Hematopoietic Stem Cell Transplantation. Pediatr. Transplant..

[43] Hayden R.T., Gu Z., Liu W., Lovins R., Kasow K., Woodard P., Srivastava K., Leung W. (2015). Risk Factors for Hemorrhagic Cystitis in Pediatric Allogeneic Hematopoietic Stem Cell Transplant Recipients. Transpl. Infect. Dis..

[44] Blazquez-Navarro A., Dang-Heine C., Wittenbrink N., Bauer C., Wolk K., Sabat R., Westhoff T.H., Sawitzki B., Reinke P., Thomusch O. (2018). BKV, CMV, and EBV Interactions and their Effect on Graft Function One Year Post-Renal Transplantation: Results from a Large Multi-Centre Study. EBioMedicine.

[45] Anderson-Smits C., Baker E.R., Hirji I. (2020). Coinfection rates and clinical outcome data for cytomegalovirus and Epstein-Barr virus in post-transplant patients: A systematic review of the literature. Transpl. Infect. Dis..

[46] Herrera S., Bernal-Maurandi J., Cofan F., Ventura P., Marcos M.A., Linares L., Cuesta G., Diekmann F., Moreno A., Bodro M. (2021). BK Virus and Cytomegalovirus Coinfections in Kidney Transplantation and Their Impact on Allograft Loss. J. Clin. Med..

[47] Aubry A., Nere M.L., Timsit S., Calvo C., Dalle J.H., Gras J., Chaix C., Gits-Muselli M., Delaugerre C., LeGoff J. (2025). Investigating Nosocomial BK Polyomavirus Infections in Pediatric Hematopoietic Stem Cell Transplantation Recipients: Challenges and Prospects. J. Infect. Dis..

[48] Rota S., Fidan K., Bozdayı G., Dalgıç A., Fidan I., Sucak G., Müderris T. (2011). Investigation of BK and JC virus DNA positivities by real-time polymerase chain reaction in the clinical samples of patients with high risk. Mikrobiyol. Bul..

[49] Rahiala J., Koskenvuo M., Sadeghi M., Waris M., Vuorinen T., Lappalainen M., Saarinen-Pihkala U., Allander T., Söderlund-Venermo M., Hedman K. (2016). Polyomaviruses BK, JC, KI, WU, MC, and TS in Children with Allogeneic Hematopoietic Stem Cell Transplantation. Pediatr. Transplant..

[50] Dybko J., Piekarska A., Agrawal S., Makuch S., Urbaniak-Kujda D., Biernat M., Rybka B., Dutka M., Sadowska-Klasa A., Giebel S. (2022). BKV Related Hemorrhagic Cystitis—An Insight into Risk Factors and Later Complications—An Analysis on Behalf of Polish Adult Leukemia Group. Cancers.

[51] Cho Y.H., Hyun H.S., Park E., Moon K.C., Min S.-I., Ha J., Ha I.-S., Cheong H.I., Ahn Y.H., Kang H.G. (2019). Higher Incidence of BK Virus Nephropathy in Pediatric Kidney Allograft Recipients with Alport Syndrome. J. Clin. Med..

[52] Delbue S., Franciotta D., Giannella S., Dolci M., Signorini L., Ticozzi R., D’Alessandro S., Campisciano G., Comar M., Ferrante P. (2020). Human Polyomaviruses in the Cerebrospinal Fluid of Neurological Patients. Microorganisms.

[53] Whiley D.M., Mackay I.M., Sloots T.P. (2001). Detection and Differentiation of Human Polyomaviruses JC and BK by LightCycler PCR. J. Clin. Microbiol..

[54] Whiley D.M., Arden K.E., Mackay I.M., Syrmis M.W., Sloots T.P. (2004). Simultaneous Detection and Differentiation of Human Polyomaviruses JC and BK by a Rapid and Sensitive PCR-ELAHA Assay and a Survey of the JCV Subtypes within an Australian Population. J. Med. Virol..

[55] Schürch W., Latour M., Barama A., Hébert M.J. (2010). Evaluation of a preemptive strategy for BK polyomavirus-associated nephropathy based on prospective monitoring of BK viremia: A kidney transplantation center experience. Transplant. Proc..

